# Different Maternal Quality Aspects Covary With the Offspring Sex Ratio and Condition in Bearded Reedlings (*Panurus biarmicus*)

**DOI:** 10.1002/ece3.72560

**Published:** 2025-11-29

**Authors:** Herbert Hoi, Ján Krištofík, Felix Knauer, Alžbeta Darolová

**Affiliations:** ^1^ Department of Integrative Biology and Evolution, Konrad Lorenz Institute of Ethology University of Veterinary Medicine Vienna Austria; ^2^ Department of Animal Ecology Institute of Zoology, Slovak Academy of Sciences Bratislava Slovakia; ^3^ Department of Integrative Biology and Evolution, Research Institute of Wildlife Ecology University of Veterinary Medicine Vienna Austria

**Keywords:** extrapair offspring, maternal effect, offspring condition, offspring sex ratio, *Panurus biarmicus*, parental quality

## Abstract

In addition to the intrinsic genetic quality of the parents, the differential allocation of maternal investment in offspring is recognized as a crucial determinant of offspring fitness. Sex allocation theory posits that females should adjust their brood sex ratio in response to the varying benefits associated with increased maternal investment in sons versus daughters. This differential allocation may occur in direct relation to the mother's condition or in relation to the attractiveness or quality of their social mate or extra‐pair mates. In this study, we investigate whether the intrinsic quality of the mother, her social or extra‐pair mate, influences the offspring sex ratio and offspring residual body mass of the socially monogamous bearded reedling ( *Panurus biarmicus* ). Our findings indicate that female intrinsic quality, in terms of tarsus length, is positively correlated with the proportion of sons produced per brood. Additionally, maternal body mass and blood sedimentation rate (a health indicator) were associated with nestling conditions, specifically residual body weight. However, the quality or attractiveness of the social mate did not affect nestling sex ratio or condition in terms of residual body mass. Furthermore, the sex ratio of extra‐pair offspring did not deviate from an equal distribution; however, nestlings fathered by extra‐pair males were in better condition than their half‐siblings fathered by the female's social mate. Our results suggest that in bearded reedlings, offspring sex is related to maternal body size, which reflects intrinsic quality, but not to maternal health or body mass.

## Graphical abstract

Our results suggest that in bearded reedlings ( *Panurus biarmicus* ), offspring sex is related to maternal body size, which reflects intrinsic quality, but not to maternal health or body mass. The sex ratio of extra‐pair offspring did not deviate from an equal distribution; however, nestlings fathered by extra‐pair males were in better condition than their half‐siblings fathered by their social mate.

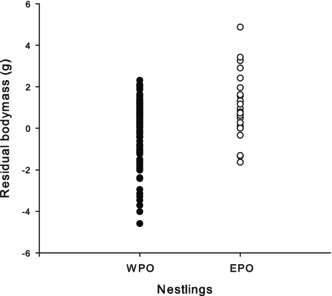

## 1 Introduction

There is a growing body of evidence highlighting the significance of early maternal investment (EMI) for offspring fitness across various animal taxa (Mousseau and Fox 1998; Gorman and Nager 2004; Maestripieri and Mateo 2009; Schoepf et al. 2022). This is particularly evident in birds, where differential allocation of maternal resources into eggs has been extensively studied (Gil et al. 1999, 2006; Gil 2003; Groothuis et al. 2005; Groothuis and Schwabl 2008; Wilson and Burley 2021). These studies indicate that EMI and early maternal effects appear to play a crucial role in determining offspring condition and fitness (Cunningham and Russell 2000; Sheldon 2000; Strasser and Schwabl 2004; McGraw et al. 2005; Rubolini et al. 2006; Bowers et al. 2017).

In addition to influencing offspring condition, there is now both correlative and experimental evidence suggesting that female birds can adaptively allocate offspring sex (Burley 1981; Arnold and Griffiths 2003; Blanco et al. 2003; Darolová et al. 2008; Arnold et al. 2016). The differential allocation of maternal investment or modification of offspring sex appears to be directly linked to the condition of the mother (e.g., Cordero et al. 2000; Whittingham and Dunn 2000; Magrath et al. 2003; Thuman et al. 2003; Correa et al. 2011; Baeta et al. 2012; Poláková et al. 2012) but may also be indirectly affected by the availability of food and other resources for the parents, particularly the mother (e.g., Frank 1990; Badyaev et al. 2002; Bell et al. 2014; Merkling et al. 2016). In line with this, the “maternal condition advantage” hypothesis posits that females should adjust the sex and sex ratio of their offspring on the basis of their own condition at the time of reproduction (Nager et al. 1999).

Moreover, the attractiveness or quality of the female's social partner (Svensson and Nilsson 1996; Ellegren and Sheldon 1997; Ligon and Hill 2010) or extra‐pair partners (Du and Lu 2010) can also be significant determinants of EMI, including sex modification although evidence regarding sex ratio modification in this relation seems to be weak (Booksmythe et al. 2017). In this context, a bias toward male or female siblings is expected to depend on which sex exhibits greater variance in reproductive success (Trivers and Willard 1973; Charnov 1982). In species where female choice is influenced by male ornaments, it can be predicted that females will invest more in and/or skew their brood sex ratio toward sons when mating with more attractive males who possess superior ornaments (Burley 1981; Ellegren et al. 1996; Svensson and Nilsson 1996).

Extra‐pair behavior is a widespread alternative tactic for increasing male reproductive success (Griffith et al. 2002; Brouwer and Griffith 2019) and is particularly significant in bird species (Petrie and Kempenaers 1998; Westneat and Stewart 2003; Cockburn 2004; Brouwer and Griffith 2019). However, active extra‐pair behavior is also frequent in females, resulting in various costs and benefits (for details see Forstmeier et al. 2014). A variety of hypotheses have been established that propose several functions, including the fertility insurance hypothesis (Sheldon 1994), avoiding inbreeding hypothesis (Kingma et al. 2013), the genetic diversity hypothesis (Westneat 1990), and the genetic compatibility hypothesis (Tregenza and Wedell 2000). Additionally, the good gene hypothesis could be a significant driving force behind female extra‐pair behavior (Birkhead and Møller 1995; Kempenaers et al. 1992; Hasselquist et al. 1996; Tregenza and Wedell 2000; Hoi and Griggio 2010), influencing EMI and driving differential resource allocation even within a brood. This strategy appears to be particularly important for lifetime monogamous females seeking to enhance the fitness of their offspring. In cases where these females are paired with “low‐quality” males, they may compensate by increasing their investment in offspring [compensatory investment hypothesis (Saino et al. 2002; Michl et al. 2005; Navara et al. 2006; Gowaty et al. 2007; Bolund et al. 2009)] or, alternatively, by engaging in extra‐pair copulations with higher‐quality males. In fact, extra‐pair copulations may be the only viable option for improving the genetic quality of their offspring (Jennions and Petrie 2000; Griffith et al. 2002). This strategy is hence crucial for enhancing offspring fitness.

According to the differential allocation hypothesis, if extra‐pair offspring (further EPO) are of higher quality, it is expected that females in lifetime pair bonds will adopt this strategy, investing more in EPO than in within‐pair offspring (further WPO) (Magrath et al. 2009; Wilson and Burley 2021).

Differences in offspring quality may arise from various factors, including the intrinsic genetic quality of the parents, and differential EMI into offspring (Petrie 1994; Neff and Pitcher 2005; Ivy 2007; Harris and Uller 2009; Kindsvater and Alonzo 2014; Sardell and DuVal 2014; Arnold et al. 2016; Haaland et al. 2017; Ratikainen et al. 2018).

The bearded reedling ( *Panurus biarmicus* ) is a species with a lifetime social monogamous mating system, usually established already soon after fledging (Hoi 1997). One important sexual trait is a male's black beard (Surmacki et al. 2015). Mate choice experiments revealed that females prefer males with longer black beards (Hoi and Griggio 2008), which was also supported by EPP (Hoi and Hoi‐Leitner 1997). Extra‐pair fertilizations are quite common in this species; almost 50% of nests contain EPO, whereby males with longer beards suffer fewer or no EPO in their own nests (Hoi and Hoi‐Leitner 1997). Additionally in a few cases males, which have been identified as those with the longest beard in a colony fathered EPO in other nests (unpublished communication). Females seem to have some control over EPP depending on their own quality (Hoi 1997; Hoi and Hoi‐Leitner 1997) as high‐quality females are more likely to be promiscuous and have EPO in their nests (Hoi and Hoi‐Leitner 1997). They initiate conspicuous chase flights with several males, representing a kind of resistance as a ploy tactic, to ensure fertilization by the best quality male not necessarily the social partner (Hoi 1997). Furthermore, beard length is negatively related to paternal investment in terms of male incubation and feeding contribution (Hoi and Hoi‐Leitner 1997). Regarding morphological measurements like tarsus‐, tail‐, wing‐ or bill length, and body weight of breeding pairs, no assortative mating was revealed (for all *p* > 0.2, *n* ≥ 27). On the basis of, this information one may expect that both sexes influence offspring fitness whereby intrinsic quality and condition of the mother should be most important.

Furthermore, a former study revealed extremely biased brood sex ratios (Darolová et al. 2009). Given that there is evidence for a maternal effect on offspring sex, we predict maternal quality parameters to be related to offspring sex ratio, whereas no direct relationship between male quality and offspring sex ratio might be expected.

On the basis of the “maternal advantage hypothesis” we would predict that residual nestling body mass is positively correlated with maternal quality in terms of body mass but negatively correlated with sedimentation rate, an indicator of maternal health, given that high sedimentation indicates lower health status.

If paternal investment is a significant determinant of offspring condition, one may expect a negative correlation between beard length of the social father and residual nestling body mass, given that beard length is negatively related to a male's incubation and feeding effort.

Assuming that EPO originates from female copulations with genetically superior or more attractive males, it can be predicted that females will skew their brood sex ratio toward sons when mating with more attractive males who possess superior ornaments. Furthermore, we may predict that these offspring will be in better physical condition (residual nestling body mass).

Assuming that females invest more in and/or skew their brood sex ratio toward sons when mating with more attractive males who possess superior ornaments (longer beards), we specifically predict a male‐biased sex ratio in EPO.

## 2 Methods

### 2.1 Study Species

The socially monogamous bearded reedlings form lifelong pair bonds already as juveniles (Koenig 1952). These birds are resident inhabitants of expansive marsh habitats throughout Europe (Glutz von Blotzheim and Bauer 1993). Bearded reedlings are known for their explosive breeding patterns, producing up to five clutches per season, with each clutch typically containing between four and seven eggs (Hoi et al. 2010). Breeding can commence as early as mid‐March and extend until mid‐August (Glutz von Blotzheim and Bauer 1993).

There is a notable plumage color dimorphism in bearded reedlings, particularly evident in the black beard stripes of males (Hoi and Griggio 2008). The length of a male's beard is an important trait for attracting females and serves as an indicator of male dominance during contests with other males (Hoi and Griggio 2008, 2011). However, beard length does not reflect an individual's actual condition (Hoi and Hoi 2001; Peiró 2021) or body size (Hoi and Griggio 2008). Males also participate in incubation and care for their offspring, with their level of parental involvement reflected by their beard length (Hoi and Hoi‐Leitner 1997). Bearded reedlings exhibit high rates of extra‐pair paternity, and instances of egg dumping and extra‐pair maternity have also been documented (Hoi and Hoi‐Leitner 1997). Interestingly, the beard length of a male appears to affect the likelihood of experiencing female extra‐pair behavior; the probability of extra‐pair offspring in a nest increases as beard length decreases (Hoi and Hoi‐Leitner 1997). Therefore, bearded reedlings provide an excellent opportunity to study EMI and a potential relationship between extra‐pair paternity and offspring condition and sex.

### 2.2 Study Area

The study was conducted in 2003 and 2005 at three sites known for their extensive reed stands and dense populations of bearded reedlings: (1) Lake Neusiedl (47°47′ N, 16°45′ E) in Austria, (2) fishponds near Veľké Blahovo (48°03′ 09.83″ N, 17°35′52.53″ E), and (3) Dolný Štál (47°57′29.31″ N, 17°44′ 21.22″ E) in Slovakia. The vegetation at these sites primarily consists of submerged reeds ( *Phragmites australis* ), interspersed with cattails ( *Typha angustifolia* and *T. latifolia* ) and sedges (*Carex* spp.). At all locations, bearded reedlings typically begin breeding at the end of March.

To investigate the relationship between EPP and nestling parameters, parentage data from a previously published study were utilized (Hoi and Hoi‐Leitner 1997). This earlier study was conducted solely at Lake Neusiedl (47°55′ 35.98″ N, 16°45′15.90″ E) and included data from 26 families in 1993 and 18 families in 1994.

### 2.3 Field Procedures

Nests were located by observing pairs during the nest‐building process and by systematically searching reed areas. In 2003 and 2005, both males and females were captured using mist nets near the nests during the nestling period. Each parent was ringed, and various measurements were taken following the same methodology as in the parentage study (Hoi and Hoi‐Leitner 1997). These measurements included tarsus length, tail length, and bill length, which were recorded using calipers to the nearest 0.1 mm, as well as body mass measured with an electronic balance to the nearest 0.1 g. Additionally, the length of the male's black beard was determined by measuring the distance from the eye to the tip of the beard (Hoi and Griggio 2008).

A small blood sample (30 to 60 μL) was also collected from the brachial vein of adult male and female bearded reedlings to assess blood sedimentation rate. This rate, which indicates the proportion of blood that sediments per hour, tends to increase in a variety of infectious and inflammatory diseases because of elevated levels of circulating fibrinogen and γ‐globulins (Gustafsson et al. 1994). To measure the sedimentation rate, capillary tubes were placed upright in a refrigerated box (4°C) for 4 h. The sedimentation rate per hour was expressed as the percentage of the tube's length containing plasma related to the length of the tube filled with blood, divided by four.

Measurements of bearded reedling nestlings were taken between 6 and 9 days of age, with all nestlings in a brood measured simultaneously. Following the same methodology as in the parentage study (Hoi and Hoi‐Leitner 1997), we measured tarsus length using calipers to the nearest 0.1 mm and body mass with an electronic balance to the nearest 0.1 g.

To compare EPO and WPO, we utilized data from the earlier study (Hoi and Hoi‐Leitner 1997). Details regarding the methods used to determine extra‐pair paternity are provided in Supplemental File 1 in Data S1, as well as in Hoi and Hoi‐Leitner (1997). We used a non‐invasive method to determine offspring sex on the basis of bill coloration between seven and 9 days of age. Male nestlings develop bright yellow bills, whereas female nestlings retain dull brownish bills (Glutz von Blotzheim and Bauer 1993). This distinction has been confirmed through molecular analyses and by tracking the nestlings to adulthood in aviaries (Darolová et al. 2009).

Following the methodology of the earlier study (Hoi and Hoi‐Leitner 1997), residual body mass relative to tarsus length was selected as an indicator of nestling condition, as it has been shown to be a reliable predictor of lipid reserves (Bachman and Widemo 1999) and survival probability (Thuman et al. 2003). Residual body mass was calculated as the residuals from an ordinary linear regression of nestling body mass against body size, as indicated by tarsus length (Green 2001). In this analysis, body mass (in grams) served as the dependent variable, whereas tarsus length (in millimeters) was the independent variable.

To compare residual body mass and within‐brood sex ratios between WPO and EPO, we utilized data and blood samples from the earlier study conducted by Hoi and Hoi‐Leitner (1997).

### 2.4 Statistics

To examine the role of male and female parent morphology, we used bill, tail, and tarsus lengths; we used body mass as a condition and blood sedimentation rate for measuring health, in relation to offspring sex and residual offspring body mass. These analyses (on the basis of our data set from 2003 and 2005 and each pair included only once) have been done separately for females and their social mates. There was no difference in clutch and brood size between study sites and respective years (for both parameters, *p* > 0.4), and there was also no difference in residual nestling body mass (*p* > 0.5). Nevertheless, location and, for the comparison between EPO and WPO, brood size were included in the models (see below).

All inferential statistical analyses were conducted in R (R Core Team 2023). Linear mixed effect models (function “gam”, Wood 2017) with percentage of sons in the brood and residual weight of the juveniles, both separated for male and female parents, were estimated. Independent variables were tarsus length, tail length, bill length, body mass, blood sedimentation rate, and, for males, additionally beard length. Missing values, mainly in the variable blood sedimentation rate, were imputed with the mean (Gelman and Hill 2006). We used data imputation not only to increase the sample size, but mostly to avoid bias due to missing cases. Especially with small sample sizes, this potential bias is hard to detect. Imputing the mean will have a neutral effect on this variable, but keeping this case in the sample will provide real information in the other variable to the model. Some individuals showed missing values in most variables. These individuals were deleted. The respective sample sizes for each analysis with and without imputation are shown in Table 1. Because of the observational nature of this study, an information‐theoretic approach was used (Burnham and Anderson 2002), which is now widely used in quantitative sciences and well accepted in observational studies. For the global models (see above), all possible variable combinations were estimated using the function “dredge” of the package MuMIn (Bartoń 2023), and for each variable, the sum of Akaike weights was calculated using the function “sw”. This is the relative variable importance (*RVI*), that is, the probability for each variable being in the model (given the global model), which explains the data best. We accepted variables with *RVI* values > 0.7 as important and constructed the final model.

**TABLE 1:** Presents parameter estimates, standard errors (SE), RVI and sample size with imputation (*N*) and in parenthesis without imputation, for the models that best explain: (a) the proportion of male nestlings in relation to maternal tarsus length, (b) residual nestling body mass in relation to maternal blood sedimentation rate and body mass, (c) the proportion of male nestlings and male parameters (no male parameter but only brood size entered this model), and (d) residual body mass of extra‐pair offspring (EPO) compared to within‐pair offspring (WPO).

Variable	Estimate	SE	RVI	*N*
(a) Intercept	−273.873	85.844	1	27 (26)
Female tarsus length	15.2	4.136	0.99	27 (26)
Deviance explained	35.1%			
(b) Intercept	2.85838	4.42980	1	28 (27)
Female blood sedimentation rate	−0.10486	0.04242	0.81	28 (27)
Female body mass	0.45482	0.19837	0.75	
Deviance explained	29.1%			
(c) Intercept	2.85838	4.42980	1	
Brood size	0.2461	0.1156	0.81	30 (24)
Deviance explained	13.91%			
(d) Intercept	0.1298	0.1301	1	144
EPO/WPO	0.8680	0.3004	0.97	
Deviance explained	5.6%			

In a second approach, we used the parentage data set from 1993 and 1994; we estimated the residual weight of 143 juveniles on the basis of extra‐pair paternity, sex, and brood size. Nest identity has been used in a General Additive Model (Wood 2017) as a random effect and brood size as a spline. Extra‐pair paternity and sex are dichotomous variables.

Finally, when examining the variation in the proportion of sons/nest in relation to paternity (EPO and WPO), we compared a model with the observed sex ratio with a model with an equal sex ratio (see Burnham and Anderson 2002).

The only potentially harmful techniques employed in this study were marking, measuring, and blood sampling of adult birds and marking and measuring of nestling bearded reedlings. Adult birds were captured using mist nets, and no birds were killed or injured during this process. Additionally, no male or female deserted their territory or nest (brood) as a result of the mist‐netting. The mist nets were continuously monitored visually, and captured birds were promptly removed. After handling, which included ringing, taking body measurements, and blood sampling, the birds were immediately released.

To gather data on nestlings, we searched for nests during the construction phase, prior to and during egg laying, allowing us to estimate the age of the nestlings. Nestlings were ringed and measured, but this was not done until they were at least 6 days old, typically between 6 and 9 days. The risk of nestlings escaping from the nest increases significantly after they reach 9 days of age. Blood samples from nestlings were only collected in the earlier study (Hoi and Hoi‐Leitner 1997) for parentage analysis, for which the necessary permits were already obtained.

During these procedures, half of the nestlings were always left in the nest to minimize unnecessary disturbance. Furthermore, no brood was predated at least 6 days following the nest inspection and treatment, indicating that our activities did not increase the risk of subsequent nest predation. All necessary permits for this work were granted by the Amt der Burgenländischen Landesregierung (5‐N‐A‐1007‐177‐2003; 5‐N‐1007/248‐2005) and the Ministry of the Environment of the Slovak Republic (2269/688/04‐5.1.pil).

## 3 Results

### 3.1 Parent Quality, Offspring Sex Ratio, and Nestling Condition

Our results, on the basis of an information‐theoretic approach, reveal that the tarsus length of the mother is a highly important predictor of offspring sex ratio (RVI = 0.99, Table 1a). No other morphological parameters, such as tail and bill length, were included in the best model (see Supplemental File 2 in Data S1). Specifically, the proportion of male offspring is positively correlated with female tarsus length (Table 1a). Additionally, maternal body mass and blood sedimentation rate did not enter the best model, which indicates that they do not contribute to explaining the variance in offspring sex ratio.

When examining nestling condition in terms of residual nestling body mass, our results indicate that maternal health, as measured by blood sedimentation rate (RVI = 0.81) and body mass (RVI = 0.75), is an important predictor of residual nestling body mass (Table 1b). The parameter estimates suggest a positive relationship between maternal body mass and residual nestling body mass, whereas the relationship with maternal blood sedimentation rate is negative. No morphological parameters of the mother were included in the best model for residual nestling body mass (Supplemental File 3 in Data S1).

In terms of the relationship between male variables and the proportion of male offspring, our results show that no male variable (morphological, body mass, or health) was included in the best model (Supplemental File 4 in Data S1). However, only brood size is related to the proportion of sons (RVI = 0.81). The positive parameter estimate suggests that the proportion of sons increases with brood size (Table 1c). Furthermore, no male or other variables were included in the model concerning the residual body mass of the nestlings (Supplemental File 5 in Data S1).

### 3.2 Sex Ratio and Residual Body Mass of EPO and WPO

When examining EPO and WPO, we compared the model with the observed sex ratio with a model with an equal sex ratio. The deviations from an equal sex ratio were negligible, given that EPO consisted of 11 sons and 16 daughters (deltaAICc = 0.61784) and WPO comprised 48 sons and 68 daughters (deltaAICc = 3.4655).

Furthermore, no difference was detected in the proportion of males between EPO and WPO (deltaAICc = 2.0502).

**FIGURE 1:**
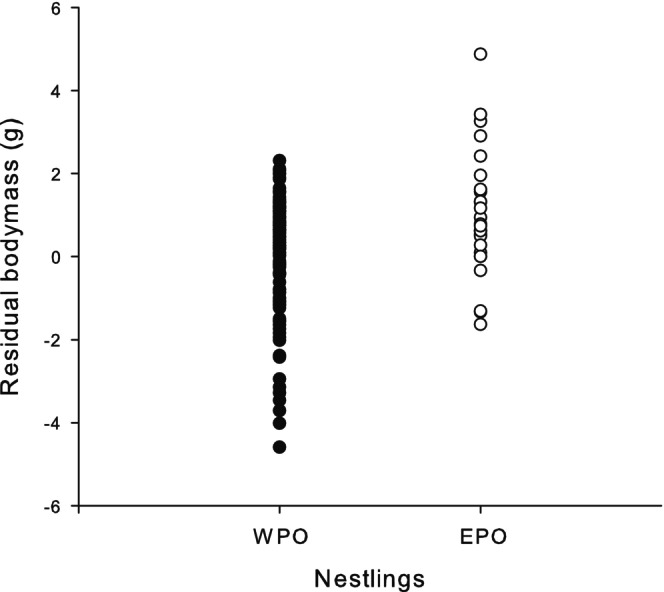
Residual nestling body mass (g), not explained by tarsus length (mm), for within‐pair offspring (WPO, *N* = 115) and extra‐pair offspring (EPO, *N* = 27).

Regarding nestling condition, our results indicate that residual body mass is strongly related to offspring origin (WPO or EPO) (RVI = 0.97, Table 1d). The positive parameter estimate suggests that nestlings resulting from extra‐pair paternity are in better condition than their within‐pair siblings (Figure 1). No other variables (sex, brood size, or nest identity) were included in the final model, which indicates that they had no important effect (Supplemental File 6 in Data S1).

## 4 Discussion

Our results support a relationship between indicators of female quality and both the sex and condition of their offspring but not male quality. This finding aligns with the “maternal condition advantage” hypothesis, which predicts that females should adjust the sex ratio of their offspring on the basis of their own body condition at the time of reproduction, leading to a positive correlation between maternal quality and the proportion of sons (Trivers and Willard 1973; Nager et al. 1999).

In this context, a rather novel finding is that maternal quality encompasses various aspects, including both current body condition and intrinsic quality. It is well established that body size is a reliable indicator of an individual's intrinsic quality (Andersson 1994; Thessing and Ekman 1994; Searcy et al. 2004; Lislevand et al. 2007; Olson et al. 2009; Labocha and Hayes 2012). Consistent with this, the intrinsic quality of bearded reedling mothers, as determined by body size (specifically tarsus length), is positively correlated with the proportion of sons in their brood. Specifically, larger females with longer tarsi tend to produce a higher proportion of sons.

Only a few studies have so far documented a similar relationship between female body size and the proportion of sons, conducted, for example, on American kestrels ( *Falco sparverius* ) (Wiebe and Bortolotti 1992), Japanese barn swallows ( *Hirundo rustica gutturalis* ) (Arai et al. 2018), and house martins ( *Delichon urbica* ) (Zielińska et al. 2010). Besides, a sex ratio effect, an influence of female body size on offspring has also been observed in Tengmalm's owls ( *Aegolius funereus* ), where female body size, but not male body size, affected reproductive success (Kouba et al. 2020). Additionally, there is evidence suggesting that female body size may impact extra‐pair paternity, for instance, in the collared flycatcher ( *Ficedula albicollis* ), larger females were less likely to produce mixed‐paternity broods (Rosivall et al. 2009).

In addition to female body size, maternal actual body mass and blood sedimentation rate are other important aspects related to nestling condition. Our results indicate that heavier female bearded reedlings tend to produce offspring in better condition, whereas females with higher blood sedimentation rates, suggesting poorer health, produce nestlings in worse condition, characterized by lower residual body mass.

Similarly, in blue tits ( *Cyanistes caeruleus* ), a stable positive relationship has been observed between female body condition (measured as residual body mass) and both nestling body mass and growth (Henderson et al. 2014). However, the correlation between maternal condition and a male‐biased brood sex ratio was much weaker (Henderson et al. 2014). In house wrens ( *Troglodytes aedon* ), a positive relationship between female condition (residual body mass) and a male‐biased sex ratio has also been documented (Whittingham et al. 2002). Nager et al. (1999) experimentally demonstrated the influence of maternal condition on offspring sex ratio in lesser black‐backed gulls ( *Larus fuscus* ).

Conversely, Kilgour et al. (2022) found no relationship between parental body condition and offspring sex ratio in starlings ( *Sturnus vulgaris* ), whereas Korpimäki et al. (2000) reported a negative relationship between the proportion of males in the brood and the condition of both female and male parents in kestrels ( *Falco tinnunculus* ). These varying results may stem from the fact that maternal condition can be influenced by different environmental factors, such as parasite burden, food availability, or weather conditions (Owen et al. 2010; Labocha and Hayes 2012; McCloy and Grace 2023), which in turn may affect parental performance.

In conclusion, different female aspects may influence different aspects of their offspring. In our reedlings, a female's actual condition and intrinsic quality (body size) are two attributes influencing different aspects of their offspring. However, the role of female quality traits may be masked. The mixed results found in this respect suggest that there are probably other, for example, external influential factors. Thus, in conclusion, the role of various female quality traits warrants further investigation.

When examining the same quality parameters for males, several studies have found a significant effect of male quality on offspring sex ratio and condition. For instance, Abroe et al. (2007) identified a relationship between brood sex ratio and male size but not ornament extension (mask dimension) in common yellowthroats ( *Geothlypis trichas* ). Similarly, Kölliker et al. (1999) demonstrated that in great tits ( *Parus major* ), the proportion of sons in the brood increased significantly with male tarsus length. Additionally, a link was found between the dimension of ornamental traits, such as plumage color, and sex ratio (Ellegren et al. 1996; Bowers et al. 2013; Cantarero et al. 2018). These findings support the “differential sex allocation” hypothesis (Burley 1981; Charnov 1982), which suggests that females mated with attractive males are more likely to produce sons. However, in our study, we found no evidence of a relationship between any male parameters, such as body size, body mass, and sedimentation rate. Why relationships exist in one sex (females) but not in the other (males) could partly reflect that there is no assortative mating in this species. However, this does not represent a causal explanation. This missing relationship also concerns male attractiveness as indicated by the length of the black beard stripe (see Supplemental File 4 in Data S1), which has been shown to be an indicator of male quality and status (Hoi and Hoi 2001) and is considered the most important trait in female choice (Hoi and Griggio 2008). Also, Booksmythe et al. (2017) found no experimental study where mothers adjust the offspring sex ratio in response to a generally preferred male phenotype. In conclusion, there is no obvious relationship between male beard length and offspring sex ratio or nestling condition (residual body mass), and consequently, no evidence that the attractiveness of the social partner influences the differential allocation rules of female bearded reedlings. However, male beard length is negatively correlated with paternal investment, specifically the proportion of time spent incubating and feeding (Hoi and Hoi‐Leitner 1997), which may mask differential allocation by the female. One possible explanation is that less attractive or genetically inferior males compensate for their deficits by increasing their investment in offspring care.

Differential sex allocation, particularly in favor of a male‐biased offspring sex ratio, may be more pronounced in EPO when extra‐pair fathers are more attractive (Ellegren et al. 1996; Sheldon et al. 1997; Griffith et al. 2002) and genetically superior. Several studies have indeed found that, in line with this theory, offspring fathered by extra‐pair sires are more likely to be male, as observed in blue tits (Kempenaers et al. 1997, but see Leech et al. 2001), red‐capped robins ( *Petroica goodenovii* ) (Dowling and Mulder 2006), red‐backed shrikes ( *Lanius collurio* ) (Schwarzová et al. 2008), and house wrens ( *Troglodytes aedon* ) (Johnson et al. 2009). However, other studies have reported no influence of extra‐pair paternity on brood sex ratio, including research on scarlet rosefinches ( *Carpodacus erythrinus* ) (Poláková et al. 2012), black swans ( *Cygnus atratus* ) (Kraaijeveld et al. 2007), blue tits (Leech et al. 2001), collared flycatchers (Sheldon and Ellegren 1996), fairy martin ( *Petrochelidon ariel* ) (Magrath et al. 2002), and barn swallows ( *Hirundo rustica* ) (Costanzo et al. 2018).

Extra‐pair paternity is relatively common in bearded reedlings and is negatively correlated with beard length (Hoi and Hoi‐Leitner 1997). Furthermore, the social rank of the female's partner, which is typically associated with beard length (Hoi and Griggio 2008), has been found to positively correlate with her extra‐pair solicitation behavior (Hoi 1997). We identified five extra‐pair males, and in all cases, the extra‐pair male had the longest beard in the respective colony (unpublished data). Therefore, we would expect beard length to be an important predictor of female extra‐pair behavior, suggesting that male bearded reedlings may exhibit greater variance in reproductive success because of the differences in ornament features. Despite this, our results did not reveal a male bias when examining EPO or WPO (Supplemental File 6 in Data S1). However, our sample size in this regard was relatively small. One reason for the lack of a detectable relationship between offspring sex ratio and extra‐pair paternity in several studies may be the inherently small sample size of EPO. Additionally, many studies on sex ratios have examined them at the individual level rather than at the brood level (Johnson et al. 2009). The ability of female birds to manipulate or even identify which of their eggs are fertilized by extra‐pair partners may be limited, given the chromosomal nature of sex determination in birds (Krackow 1995; West and Sheldon 2002; Johnson et al. 2009).

In addition to the small sample size, another explanation for the equal distribution of EPO sex may be that females are unable to determine which male's sperm fertilizes which egg, or that the mechanisms for sex determination are not sufficiently refined. Consequently, when females engage in extra‐pair copulations, they may only be able to randomly bias the sex of their offspring within the brood, thereby increasing the likelihood that at least some EPO are male. This could result in a male bias at the brood level (brood sex ratio) without necessarily affecting the sex ratio at the individual level (i.e., among EPO).

However, in addition to paternity, other factors—such as social, physiological, or environmental influences—may simultaneously affect offspring sex in ways that do not align strictly with paternity, potentially weakening the relationship between sex and paternity (Johnson et al. 2009).

When examining parental quality in relation to nestling condition (residual body mass) and EPO, we found evidence that the quality of the mother but not of the social father impacts the residual body mass of the offspring, which may reflect their overall condition (as discussed earlier). Nestlings resulting from extra‐pair copulations were in better condition than within‐pair offspring (WPO) (Figure 1). This finding could be attributed to the superior genetic quality of the extra‐pair father (Neff and Pitcher 2005) or to differential maternal investment in offspring sired by these extra‐pair males (Magrath et al. 2009; Ferree et al. 2010; Wilson and Burley 2021).

Several studies have reported differences in condition or size between EPO and WPO (Freeman‐Gallant et al. 2006; Costanzo et al. 2018; Zhang et al. 2024), whereas others have not found such differences (Kleven and Lifjeld 2004; Rosivall et al. 2009; Ortega et al. 2023). Magrath et al. (2009) and Ferree et al. (2010) observed differences in their studies, but after controlling for variations in hatching order within broods, none of the observed disparities remained significant. Some studies have even suggested that EPO may be of inferior quality (Lampila et al. 2011; Sardell et al. 2012). Thus, the results regarding the condition and quality of EPO are mixed.

To clarify the causal relationship, further investigation is needed to determine whether more resources are allocated to the eggs of extra‐pair males (Cordero et al. 2001), whether females manipulate the laying order (Badyaev et al. 2002; Blanco et al. 2003; Magrath et al. 2003), or whether ambient temperature (Arct et al. 2013, 2024) affects the growth of EPO. In a previous study (Darolová et al. 2008), we demonstrated that in bearded reedlings, female offspring hatched first and had a higher initial body mass, but male offspring developed faster, offsetting the earlier hatching advantage of female nestlings.

In conclusion, although our earlier study indicated significant variation in within‐brood sex ratio (Darolová et al. 2009), our recent findings suggest that such a sex ratio bias is not a result of EPO. Instead, differences in female intrinsic quality, as indicated by tarsus length, may be responsible for the male‐biased sex ratio and the overall superior condition of offspring, particularly among EPO.

## Author Contributions

**Herbert Hoi:** conceptualization (equal), data curation (equal), formal analysis (equal), writing – original draft (lead). **Ján Krištofík:** data curation (equal), investigation (equal), methodology (equal), writing – review and editing (equal). **Felix Knauer:** data curation (equal), formal analysis (equal), writing – review and editing (equal). **Alžbeta Darolová:** conceptualization (equal), data curation (equal), investigation (equal), methodology (equal), visualization (equal), writing – review and editing (equal).

## Funding

This study was funded by the Agency for the Support of Science and Technology APVT [grant numbers 51‐00402].

## Conflicts of Interest

The authors declare no conflicts of interest.

## Supporting information


**Data S1:** ece372560‐sup‐0001‐DataS1.zip.

## Data Availability

All data included in this manuscript is uploaded as Supporting Information.
